# Surgeon Engagement with Patient-Reported Measures in Australian and Aotearoa New Zealand Bariatric Practices

**DOI:** 10.1007/s11695-022-06237-z

**Published:** 2022-08-16

**Authors:** Alyssa J. Budin, Priya Sumithran, Andrew D. MacCormick, Ian Caterson, Wendy A. Brown

**Affiliations:** 1 Department of Surgery, Monash University, The Alfred Centre, Melbourne, VIC 3004 Australia; 2 Department of Medicine, St Vincent’s Hospital, University of Melbourne, Melbourne, VIC 3000 Australia; 3 Department of Surgery, The University of Auckland, Auckland, 1142 New Zealand; 4 Counties Manukau District Health Board, Otahuhu, Auckland, 1640 New Zealand; 5 The Boden Institute, Charles Perkins Centre, The University of Sydney, Camperdown, NSW 2006 Australia; 6 Alfred Health, The Alfred Centre, Melbourne, VIC 3004 Australia

**Keywords:** Bariatric surgery, Patient-reported measures, Patient-reported outcomes, Health-related quality of life, Psychosocial health

## Abstract

**Purpose:**

Patient-reported measures are an important emerging metric in outcome monitoring; however, they remain ill-defined and underutilized in bariatric clinical practice. This study aimed to determine the characteristics of patient-reported measures employed in bariatric practices across Australia and Aotearoa New Zealand, including barriers to their implementation and to what extent clinicians are receptive to their use.

**Methods:**

An online survey was distributed to all bariatric surgeons actively contributing to the Australian and Aotearoa New Zealand Bariatric Surgery Registry (*n* = 176). Participants reported their use of patient-reported measures and identified the most important and useful outcomes of patient-reported data for clinical practice.

**Results:**

Responses from 64 participants reported on 120 public and private bariatric practices across Australia and Aotearoa New Zealand. Most participants reported no collection of any patient-reported measure (39 of 64; 60.9%), citing insufficient staff time or resources as the primary barrier to the collection of both patient-reported experience measures (34 of 102 practices; 33.3%) and patient-reported outcome measures (30 of 84 practices; 35.7%). Participants indicated data collection by the Registry would be useful (47 of 57; 82.5%), highlighting the most valuable application to be a monitoring tool, facilitating increased understanding of patient health needs, increased reporting of symptoms, and enhanced patient-physician communication.

**Conclusion:**

Despite the current lack of patient-reported measures, there is consensus that such data would be valuable in bariatric practices. Widespread collection of patient-reported measures by registries could improve the collective quality of the data, while avoiding implementation barriers faced by individual surgeons and hospitals.

**Graphical abstract:**

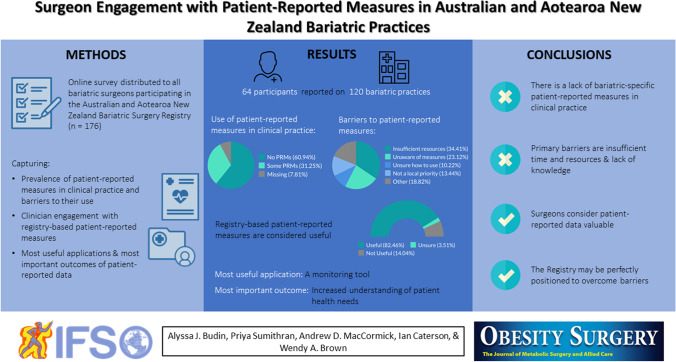

**Supplementary Information:**

The online version contains supplementary material available at 10.1007/s11695-022-06237-z.

## Introduction

The efficacy of bariatric (metabolic) surgery has been well established in the treatment of obesity and associated medical problems, with the popularity of such procedures escalating in Australia and internationally [1–4]. The rising prevalence of bariatric procedures has generated a call for improved reporting of outcomes, which to date have been inconsistent and ill-defined. A recent review identified over 1000 individual reported outcomes following bariatric procedures, most of which were only reported in a single paper [5]. This heterogeneity of reporting prevents meaningful interpretation of data and delays downstream impacts on clinical practice.

An important emerging component of reporting is the use of patient-reported measures (PRMs) to capture and quantify aspects of the patient experience and outcomes, particularly surrounding quality of life and psychosocial wellbeing [6]. In addition to traditional clinical markers such as mortality, readmission, complication rates, and weight change, PRMs describe outcomes that are often most important to patients and may provide a more sensitive measure of patient progress [7, 8]. PRMs include Patient-Reported Experience Measures (PREMs), capturing a patient’s perception of their experience of care, and Patient-Reported Outcome Measures (PROMs) capturing patient’s perception of their health and wellbeing, as well as behavioral outcomes such as physical activity and diet. PRMs are increasingly being utilized in research, registries, and clinical practice across multiple disease classifications and healthcare systems, highlighting changes in symptoms, promoting patient engagement with their treatment, aiding in clinical decision making, improving overall patient outcomes, and improving the quality of health service provision [9–12]. The capture of these factors is of particular importance in the bariatric field owing to the recognition of adverse patterns of depression, self-harm and suicidal ideation, alcohol and substance abuse, maladaptive eating behaviors, and deteriorating body image among this patient population [13–15]. Despite the utility and evident relevance of PREMs and PROMs, they are yet to become commonplace within routine bariatric clinical practice, with little to no literature describing the prevalence or types of PRMs used in bariatric practices, particularly across Australia and Aotearoa New Zealand.

The Australian and Aotearoa New Zealand Bariatric Surgery Registry (ANZ BSR) is a clinical quality and safety registry capturing clinical data for patients undergoing bariatric surgery across public and private hospitals in Australia and Aotearoa New Zealand. The ANZ BSR captures data from 89% of eligible hospitals and 82% of eligible surgeons, making it well situated to facilitate unified and consistent collection of PRMs. In aid of developing centralized PRMs, it is pertinent to first understand where and to what extent PROMs and PREMs are currently used in routine clinical practice throughout Australia and Aotearoa New Zealand. Our primary objectives were to (1) determine the characteristics and distribution of PRMs employed in bariatric practices, and (2) examine clinician engagement with the development of centralized PRMs by the ANZ BSR, and identify the most important and useful outcomes for clinical practice. It was hypothesized that a higher patient burden may impact a surgeons’ capacity to collect PRMs in clinical practice. Australia has a larger population with many more patients undergoing bariatric surgery compared to Aotearoa New Zealand, while certain states/territories within Australia (Victoria, New South Wales, and Western Australia) also have a higher patient volume than other jurisdictions. Differences between countries, and jurisdictions within Australia, as well as between surgeons with a higher number of practices or larger patient volume, were analyzed to elucidate any effect of population and/or patient burden on implementation and use of PRMs.

## Methods

### Study Design and Population

A cross-sectional survey was designed to examine current practice for PREM and PROM collection within bariatric practices across Australia and Aotearoa New Zealand, including the extent of PRM measurement within clinical practice, how and why this data was collected and subsequently used, and the opinions of survey participants as to the utility of registry-based PRM data.

The ANZ BSR captures data from 197 surgeons in 133 public and private hospitals across Australia and Aotearoa New Zealand. The majority of sites and surgeons are located in Australia (184 surgeons, 122 sites), with more populous Australian jurisdictions (Victoria, New South Wales, and Western Australia) representing a higher proportion. Bariatric procedures are predominately performed in women in private hospitals in both Australia (78.0% female; 94.5% Private) and Aotearoa New Zealand (79.5% female; 77.4% Private). The mean age for primary procedures is 42.4 years in Australia and 45.8 years in Aotearoa New Zealand with Sleeve Gastrectomy being the dominant procedure in both countries (67.6% Australia; 58.6% Aotearoa New Zealand) [16]. All surgeons actively participating in the ANZ BSR received a personalized invitation to participate in the survey (*n* = 176 at time of invitation).

### Survey Design and Administration

Survey questions were adapted from the survey developed by Morton et al. [11] and refined collaboratively within the ANZ BSR research team based on a review of data points collected by similar Clinical Quality Registry studies in both Australia and Aotearoa New Zealand, and internationally [17–20], as well as a preliminary framework for the inclusion of PRMs in Registries [21] and expert knowledge within the ANZ BSR. The survey consisted of two parts with 23 items overall. The first part had 17 items exploring characteristics of current PRM use including whether PRMs were collected in routine clinical practice, reasons PRMs were or were not collected, the instruments used, administration methods, collection frequency, and interpretation of resulting PRM data. Participants were asked to repeat all items in Part 1 of the questionnaire for each individual practice in which they operate and to identify each practice as within either the public or private healthcare sectors. The second part of the survey had 6 items gauging each participants’ interest and engagement with registry-based PRM collection and their opinions on the applications and outputs of registry-based PRM data.

Follow-up emails were sent at 1, 4, and 12 weeks following initial invitation including options for a nominated delegate to complete the survey on behalf of the surgeon, as well as to complete a short-form of the survey. The short-form survey was released 4 weeks after first invitation to invite additional participant responses. The short-form survey asked participants to divide their response into public and/or private practices, rather than providing responses for each individual practice. This simplification of the questionnaire was employed to avoid surgeons with multiple practices abandoning the survey prior to completing the questionnaire for all of their practices.

Surveys were completed via the secure online platform Qualtrics (Qualtrics, Provo, UT).

### Statistical Analysis

Surgeon responses were organized into participant responses and practice responses. Participant responses represent the collective answers provided by each surgeon, while practice responses represent the specific answers provided for an individual practice. For example, a surgeon reporting on 3 separate practices would repeat Part 1 of the questionnaire 3 times, once for each practice. This would generate 1 participant response and 3 practice responses. Partial responses of full-form surveys without a single complete practice response and short-form survey responses without a complete public or private practice response were excluded from analysis. Responses from full-form and short-form surveys were crosschecked for repetition of respondents and collapsed into individual practice responses as appropriate. Any practice that was reported by more than one participant was checked for consistency and collapsed as appropriate. If any differences were observed between responses, they were maintained as separate practice responses. This reflects multiple surgeons operating at a single center, but providing individual services which may differ in their approach.

In addition to survey responses, the number of procedures performed by each responding surgeon in the year preceding the survey was obtained from the ANZ BSR database as a measure of patient volume.

Descriptive statistics (frequencies and percentages) were reported for each participant and practice response, to each survey item. *χ*^2^ tests were used to assess differences in proportions between public and private practices as well as between countries and jurisdictions. Mann–Whitney *U* and Kruskal–Wallis tests were used to assess whether there were any differences in the number of practices or number of procedures between countries, jurisdictions, public/private practices, and PRM use. Related-samples Friedman’s analysis of variance (ANOVA) and post hoc multiple comparisons with Bonferroni correction were used to assess differences in ranked items. Qualitative data were analyzed thematically to identify any barriers to registry-based PRM administration, preferences for data collection, and utility of data in the clinical setting. Statistical analysis was performed using SPSS (IBM SPSS Statistics for Windows, Version 27), with statistical significance inferred at a *p* value of < 0.05.

## Results

### Participant Responses

Responses to the PRMs ANZ Survey were received from 69 surgeons, a response rate of 39.2% (69 of 176 invited surgeons, Table 1). Five surgeon responses contained insufficient data and were excluded from analysis, with the remaining 64 participants reporting on 120 public and private bariatric practices across Australia and Aotearoa New Zealand. The distribution of responders and practices across jurisdictions was similar to that of the ANZ BSR [16], and there was no difference between responders and non-responders by country, jurisdiction, or procedure volume (*p* > 0.05).

**Table 1:** Public and private Australian and Aotearoa New Zealand responders, non-responders, and practice responses

	Responders		Non-responders		Practices	
	*n*	%	*n*	%	*n*	%
**Total**	**69**		**107**		**120**	
Public only	4	5.8			25	20.8
Private only	39	56.5			95	79.2
Public and private	18	26.1				
*Missing *^*a*^	*8*	*11.6*				
**Australia**	**62**	**89.2**	**103**	**96.3**	**107**	**89.2**
Australian Capital Territory	0	-	1	1.0	0	-
New South Wales	16	25.8	28	27.2	22	18.3
Northern Territory	2	3.2	0	-	3	2.5
Queensland	12	19.4	20	19.4	23	19.2
South Australia	3	4.8	6	5.8	4	3.3
Tasmania	1	1.6	1	1.0	2	1.7
Victoria	17	27.4	38	36.9	34	28.3
Western Australia	11	17.7	9	8.7	19	15.8
**Aotearoa New Zealand**	**7**	**10.8**	**4**	**3.7**	**13**	**10.8**
North Island	7	100	2	50.0	13	10.8
South Island	0	-	2	50.0	0	-

% indicates column percentages

^a^8 participant responses were incomplete; 3 did not provide complete practice information, and 5 were excluded from analysis

Overall, most participants reported no collection of any PRMs (39 of 64; 60.9%, Table 2). Those that did collect measures most commonly collected only PROMS (9 of 64; 14.1%), as opposed to only PREMs (4 of 64; 6.3%) or both PREMs and PROMs (7 of 64; 10.9%). PRMS were predominantly collected by pen and paper (6 of 20; 30.0%), administered by a clinician or nurse (4 of 20; 20.0%), or captured electronically (4 of 20; 20.0%). Seventeen participants (26.6%) reported they had previously collected a PRM that had since been discontinued. The primary reasons for discontinuation were insufficient resources to collate and analyze PRMs (6 of 17; 35.3%) and too many questions in the measure (2 of 17; 11.8%). There were no significant differences in the collection of PRMs between participants from Australian and Aotearoa New Zealand, across jurisdictions, or based on the number of practices or the number of procedures performed by participants (*p* > 0.05).

**Table 2:** Collection of PRMs by Australian and Aotearoa New Zealand bariatric surgeons, operating in public, private, or both public and private bariatric practices

	Participants	No PRMs		PREMs only		PROMs only		PREMs and PROMs	
	*n*	*n*	%	*n*	%	*n*	%	*n*	%
**Total**	**64**	**39**	**60.9**	**4**	**6.3**	**9**	**14.1**	**7**	**10.9**
Public only	4	1	25.0	0	-	0	0.0	3	75.0
Private only	38	26	68.4	4	10.5	6	15.8	2	5.3
Public and private	17	12	70.6	0	-	3	17.6	2	11.8
*Missing *^*a*^	*5*								
**Australia**	**52**	**36**	**92.3**	**3**	**5.8**	**8**	**15.4**	**5**	**9.6**
Australian Capital Territory	0	0	-	0	-	0	-	0	-
New South Wales	12	8	66.7	2	16.7	2	16.7	0	-
Northern Territory	2	1	50.0	0	-	0	-	1	50.0
Queensland	9	7	77.8	0	-	2	22.2	0	-
South Australia	3	2	66.7	0	-	0	-	1	33.3
Tasmania	1	0	-	0	-	0	-	1	100
Victoria	15	10	66.7	1	6.7	2	13.3	2	13.3
Western Australia	10	8	80.0	0	-	2	20.0	0	-
**Aotearoa New Zealand**	**7**	**3**	**42.9**	**1**	**14.3**	**1**	**14.3**	**2**	**28.6**

% indicates row percentages

Abbreviations: *PRMs* patient-reported measures, *PREMs* patient-reported experience measures, *PROMs* patient-reported outcome measures

^a^5 participants did not provide complete PRM data

### Practice Responses

#### Patient-Reported Experience Measures

The majority of practices reported no collection of PREMs (102 of 120 practices; 85.0%), with no difference between Australian and Aotearoa New Zealand (86.9% vs 69.2%, *p* = 0.202) or public and private practices (76.0% vs 87.4%, *p* = 0.271). The most common reasons for not collecting PREMs were insufficient staff time or resources (34 of 102; 33.3%), being unaware of available PREMs (24 of 102; 23.5%), PREMs not a local priority (16 of 102; 15.7%), and unsure of how to collect or use PREM data (8 of 102; 7.8%). Other reasons for not collecting PREMs included PREMs not being considered useful or impactful and that PREMs either had been discontinued or were being planned for implementation (Table 3).

**Table 3:** Reasons for collecting and not collecting PREMs and PROMs in public and private bariatric practices

	Total	%	PREMs	%	PROMs	%
**Reasons PRMs were collected **^**a**^	**51**		**18**		**33**	
Mandated by health service	5	9.8	3	16.7	2	6.1
Auditing clinical practice	13	25.5			13	39.4
Monitoring and improving service/practice	16	31.4	16	88.9		
Screening surgical candidates	9	17.6			9	27.3
Directly informs clinical care	8	15.7			8	24.2
Research purposes	11	21.6	6	33.3	5	15.2
Collected but not used	1	2.0	0	-	1	3.0
Other	6	11.8	2	11.1	4	12.1
**Reason PRMs were not collected **^**a**^	**186**		**102**		**84**	
Insufficient staff time or resources	64	34.4	34	33.3	30	35.7
Not aware of available PRMs	43	23.1	24	23.5	19	22.6
Unsure how to collect or use data	19	10.2	8	7.8	11	13.1
Not a local priority	25	13.4	16	15.7	9	10.7
Not regarded as useful or impactful	9	4.8	6	5.9	3	3.6
Previous PRMs discontinued	8	4.3	4	3.9	4	4.8
Planning to implement PRMs	10	5.4	6	5.9	4	4.8
Other	8	4.3	4	3.9	4	4.8

% indicates column percentages

Abbreviations: *PRMs* patient-reported measures, *PREMs* patient-reported experience measures, *PROMs* patient-reported outcome measures

^a^Multiple answers possible

Those practices that did collect PREMs typically collected them once (61.5%), annually (15.4%), or ad hoc/as needed (23.1%) for the purposes of monitoring and improving quality of service (16 of 18; 88.9%) and for research (6 of 18; 33.3%) (Table 3). The PREMs used were generally practice-specific (11 of 18; 61.1%), or unknown to the respondent, while the Patient Satisfaction Questionnaire (PSQ) was used in 4 of 18 practices (22.2%).

#### Patient-Reported Outcome Measures

The majority of practices reported no collection of PROMs (84 of 117; 71.8%), with no difference between Australian and Aotearoa New Zealand (71.2% vs 76.9%, *p* = 0.913) or public and private practices (62.5% vs 74.2%, *p* = 0.379). The primary reasons PROMs were not collected included insufficient time or resources (30 of 84; 35.7%), being unaware of available PROMs (19 of 84; 22.6%), unsure how to collect or use PROM data (11 of 84; 13.1%), and PROMs not being a local priority (9 of 84; 10.7%) (Table 3).

PROMs were mostly commonly collected pre- and post-operatively (10 of 33; 30.3%) or post-operatively only (10 of 33; 30.3%). When used pre-operatively, PROMs were collected only once (100%), while post-operatively they were collected once (9 of 20; 45.0%), at each clinic appointment (3 of 20; 15.0%) or at regular time points (3 of 20; 15.0%). Two validated instruments were routinely used: the Beck Depression Inventory (BDI) in 7 of 33 practices (21.2%) and the SF-36 in 8 of 33 practices (24.2%). Most commonly, bespoke, practice-specific PROMs were used (16 of 33 practices; 48.5%), measuring outcomes such as weight, changes in associated medical problems, and quality of life. The primary purpose for collecting these PROMs was to audit clinical practice (13 of 33; 39.4%), screen surgical candidates (9 of 33; 27.3%), directly inform clinical care (8 of 33; 24.2%), and for research purposes (5 of 33; 15.2%) (Table 3).

### Participant Engagement with Registry-Based PRMs

The majority of respondents believed that Registry-based collection of PRM data would be useful (47 of 57; 82.5%), eight were unsure (14%), and two believed it would not be useful (3.5%). For those who answered no or unsure, reasoning provided was primarily regarding a lack of empirical evidence as to their utility, concern for their ability to change clinical practice, and previous experience with PRMs that were not useful.

Respondents were asked to rank five potential applications of registry-based PRMs from most to least useful and six potential outcomes of registry-based PRMs from most to least important. The results of the Friedman tests indicated there was a statistically significant difference in rank across the applications (*p* < 0.001) and outcomes (*p* < 0.001). The mean and median ranks for each application and outcome are listed in Table 4.

**Table 4:** Mean and median ranks for applications and outcomes of registry-based PRMs

	Mean	Median	IQR
**Applications of registry-based PRMs**			
A monitoring tool	2.35	2.00	1.00–4.00
Evaluating effectiveness of care	3.19	3.00	1.00–6.00
Promoting shared decision making	3.48	3.00	2.00–5.00
A screening tool	3.90	4.00	3.00–5.00
A decision aid	3.92	4.00	2.25–5.00
Facilitating communication among MDTs	4.17	4.50	3.00–6.00
**Outcomes of registry-based PRMs**			
Increased understanding of patient health needs and QoL	2.27	2.00	1.00–3.00
Increased reporting and recognition of symptoms	2.38	2.00	1.00–3.00
Enhanced patient physician communication	2.76	3.00	2.00–3.50
Reduced strain on staff time/resources	3.33	4.00	2.00–5.00
More actions taken based on PRO data	4.27	5.00	4.00–5.00

Abbreviations: *PRMs* patient-reported measures, *MDTs* multi-disciplinary teams, *QoL* quality of life, *PRO* patient-reported outcome

Respondents indicated reservations about the validation of PRM utility in clinical practice, although many highlighted the importance of patient-reported data to both clinicians and patients, emphasizing the need for the PRM to be easy to complete, short, and delivered electronically, to facilitate widespread rollout, citing the burden PRM administration could have, particularly on smaller practices.

## Discussion

This survey captured the characteristics and distribution of patient-reported measures (PRMs) employed within a representative sample of bariatric practices across Australia and Aotearoa New Zealand. The majority of participants reported no collection of any PRMs, with time and resources being the primary barrier to their use in clinical practice. PROMs were collected more often than PREMs; however, the majority of both PREMs and PROMs collected were non-specific or non-validated measures, collected at only one time point pre- or post-surgically. There were no differences found in the use of PREMs or PROMs between countries, jurisdictions, practice number, or patient volume, highlighting the lack of PRM implementation is ubiquitous across the study population and was not dictated by patient burden. Despite a clear lack of current uptake, participants agreed PRM data would be useful, highlighting the most valuable application would be as a monitoring tool, and the most important outcomes to be increased understanding of patient health needs and quality of life, increased recognition and reporting of symptoms, and enhanced patient-physician communication.

The PRMs identified within our survey are inconsistent and varied, with a notable lack of validated, reliable instruments specific to the experiences and outcomes of bariatric patients. A recent systematic review highlighted this variability, identifying 68 different validated measures across 86 bariatric surgery studies [22]. As in our survey, the Medical Outcomes Short Form SF-36 (36%) and Beck Depression Inventory (20%) were the most frequently used outcome measures, with the following nine most common measures also being generic or aspect-specific [22]. Experience measures were similarly lacking, with the generic Patient Satisfaction Questionnaire (22%) being the most common validated measure in our study. The majority of practices using PREMS used bespoke practice-specific questions (61%), particularly focused on patient satisfaction. The application of generic measures in specific disease or treatment populations represents a key barrier to widespread PRM uptake, as they fail to generate actionable data [22–24]. Several survey respondents highlighted this in their hesitancy to endorse PRMs, citing a concern for their ability to impact clinical practice, and the importance of the data having clinical meaning. Despite the need for specific patient-reported data and the observed heterogeneity within the literature, there remains no consensus on the most appropriate measures to be used in people with severe obesity and those undergoing bariatric procedures, limiting their uptake in clinical practice [5, 22, 24–26]. This does not, however, diminish the utility of patient-reported data, with only 20% of primary care physicians in the USA indicating that PROs were unhelpful [27], mirrored in our survey with 3.5% of respondents believing PRM data not to be useful and 14% being unsure. These findings, alongside the multitude of surgeons utilizing bespoke questions to capture patient experiences and outcomes, highlight that patient-reported data is perceived to be of clinical benefit.

In our survey, respondents identified a monitoring tool to be the most useful application of patient-reported data. PRM monitoring tools provide regular, ongoing feedback to clinicians, allowing them, and their patients, to reflect on their progress and respond to changes. Studies of PRMs used as monitoring tools found they increased early detection and discussion of problems by clinicians, with an impact on subsequent clinician intervention provided the PRM was specific to the condition or treatment being assessed [12, 28]. Despite a low usefulness rating in our survey, using PRMs as screening or diagnostic tools has been well established and is often highly valued by primary care physicians and GPs[28, 29]. Screening tools in a bariatric context are most applicable to pre-surgical evaluations and may be less useful than long-term monitoring, particularly given the tendency of adverse mental health effects to develop 2–4 years post-operatively [15, 30, 31].

In Australia and Aotearoa New Zealand, the bi-national Bariatric Surgery Registry may be perfectly situated to harmonize the heterogeneous collection of PRMs, while avoiding implementation barriers faced by individual practices or hospitals. PRMs are increasingly being incorporated into registries across Australia and internationally to understand the trajectory of patients’ symptom burden and quality of life over the course of disease and/or treatments [17, 20, 21]. The large database generated by registry-based PRM collection would facilitate summative results of the different bariatric procedure types, generate outputs specific to a patients’ demographics, aid in population health-management, and generate an avenue for comparative effectiveness evaluations [23, 26, 32, 33]. Continuing work towards a bariatric PRM core set and the appropriate implementation of PRMs in registries may pave the way for consistent, long-term collection of PRM data in Australia and Aotearoa New Zealand, and internationally [20, 21].

### Strengths and Limitations

This study was strengthened by collecting multiple responses from each participant and differentiating between public and private practices. This enabled the expansion of data beyond the individual and facilitated important insight into any disparities between the public and private sectors. This is also the first review of PRM use in bariatric practices in the Australian and Aotearoa New Zealand context, providing an important baseline to guide continued development and implementation of PRMs, ensuring the results are relevant and meaningful to clinical practice.

The response rate of 39% is a considerable limitation of this study, and the results may not be reflective of the entire population of surgeons across Australia and Aotearoa New Zealand. However, the response rate is comparable to similar surveys of surgeons in Australia and Aotearoa New Zealand [34–37] as well as research exploring the response rates of physicians to web-based surveys in which 35% of physicians responded, and surgeons were less likely to respond than other medical specialties [38, 39]. In addition, the proportion of respondents from Australia and Aotearoa New Zealand, and across jurisdictions, is representative of those contributing to the Bariatric Surgery Registry, with no significant differences identified between responding and non-responding surgeons, reducing the likelihood of nonresponse bias. As work continues, validation of these results will be important to facilitate the implementation of PRMs in practices across Australia and Aotearoa New Zealand.

In completing the short-form survey, surgeons reported their practices as only public or private, and any duplication of specific practice sites cannot be accounted for. Finally, the results are specific to the Australian and Aotearoa New Zealand context and may not be applicable to international populations; however, the paucity of bariatric-specific PRM collection [5, 22], barriers to PRM implementation [29, 33, 40], and consensus regarding the potential utility of PRM data is mirrored in multiple international studies [23, 24, 27].

## Conclusions

There is an evident lack of patient-reported measures used in Australian and Aotearoa New Zealand bariatric practices. The majority of practices did not collect any PRMs, with the primary barrier to PRM uptake being a lack of knowledge regarding available PRMs, or insufficient time and resources to implement them. Those that do employ PRMs most commonly use non-specific, or non-validated measures, collected at only one time point. Despite this, surgeons indicated that registry-based PRM data would be useful, highlighting the most useful application would be as a monitoring tool, and the most important outcomes to be increased understanding of patient health needs and quality of life, increased recognition and reporting of symptoms, and enhanced patient-physician communication.

## Supplementary Information

Below is the link to the electronic supplementary material.

Supplementary file1 (DOCX 462 KB)
