# Supplementary material for: Surgeon Engagement with Patient-Reported Measures in Australian and Aotearoa New Zealand Bariatric Practices
Source: Obes Surg. 2022 Aug 16;32(10):3410–8. doi: 10.1007/s11695-022-06237-z (PMC9532331; doi:10.1007/s11695-022-06237-z)
Supplement: Supplementary file 1 — Supplementary file1 (DOCX 462 KB) [file 11695_2022_6237_MOESM1_ESM.docx]

**The Use of Patient-Reported Measures in Australian and Aotearoa New Zealand Bariatric Centres**

In this survey we will ask you to detail your use of patient-reported experience measures (PREMs) and patient-reported outcome measures (PROMs) including which questionnaires are used, the method and frequency of administration, and how the data is analysed.

We will also ask for your judgement on the most useful and important outcomes and applications of registry-based PRM collection. The data you provide will guide the continued development of BSR PRMs to ensure the final result is one of utility and meaning in the clinical context. The results of this survey may lead to publications and/or be used in future presentations by the BSR.

PLEASE NOTE:

Throughout this survey we will refer to patient-reported experience measures (PREMs), and patient-reported outcome measures (PROMs) individually, as well as patient-reported measures (PRMs) which encompasses both PREMs and PROMs.

Name of person completing the survey:

| First Name: | Surname: |
| --- | --- |

Are you completing this survey on someone else’s behalf?

|  | No | | |  |  |
| --- | --- | --- | --- | --- | --- |
|  | Yes | (If yes, please write their name below) | |  |  |
| First Name: | | | | Surname: | |

**SECTION ONE** (please repeat this section for each practice in which you operate)

Name of surgery / rooms / clinic: _______________________________________________________

Does this response relate to a public or private practice?

|  | Public | |
| --- | --- | --- |
|  | Private |  |

1. Does your surgery/rooms/unit currently collect any patient-reported experience measures (PREMs) for bariatric patients as part of routine clinical practice?

Note: PREMs = questionnaires capturing the patient’s observations and perceptions of their healthcare experience. This may include questions about information provision, access to services, quality and safety, services and equipment, and aspects of the patient-clinician interaction.

|  | None If none, please go to question 2 | |
| --- | --- | --- |
|  | Yes, specifically for bariatric patients | If yes, please skip to question 3 |
|  | Yes, for all patients |  |

1. What is the main reason PREMs are not collected?

|  | Insufficient staff time or resources to follow-up on PREMs |
| --- | --- |
|  | Not aware of available PREMs |
|  | Unsure how to collect or use PREM data |
|  | PREMs not a local priority |
|  | PREMs not regarded as useful or impactful |
|  | Previously used PREMs have been discontinued |
|  | Planning to implement PREMs |
|  | Other *(please specify)* |
|  |  |

Please skip to question 6

1. What are the main purposes for collecting PREMs?

(Please select all that apply)

|  | Mandated by health service |
| --- | --- |
|  | Monitoring and improving quality of service provision |
|  | Research purposes |
|  | Collected but not used |
|  | Other *(please specify)* |
|  |  |

1. Which PREM questionnaires does your surgery/rooms/unit currently use?

(Please select all that apply)

|  | AHPEQS (Australian Hospital Patient Experience Question Set) | | |  |
| --- | --- | --- | --- | --- |
|  | PSQ (Patient Satisfaction Questionnaire) | | |  |
|  | HCAHPS Survey (Hospital Consumer Assessment of Healthcare Providers and Systems) | | | |
|  | PEQ / PPE-15 (Picker Patient Experience Questionnaire) | | |  |
|  | PEECH (Patient Evaluation of Emotional Care during Hospitalisation) | | |  |
|  | Surgery/Rooms/Unit-specific PREM *(please outline what is assessed/measured)* | | |  |
|  |  |  |  |  |
|  | Other *(please specify)* |  |  |  |
|  |  | | |  |

1. How frequently do you collect PREMs?

|  | Once (e.g. on discharge, 1 week post-op)  *(Please specify time point)* ..……………………………………………………………………………… | | |
| --- | --- | --- | --- |
|  | At defined time points (e.g. every 6 weeks, 6 months, annually)  *(Please specify)* …………………………………………………………………………………………………. | | |
|  | At each clinic appointment |  |  |
|  | Ad-hoc / as needed |  |  |
|  | Other *(please specify)* |  |  |
|  |  | | |

1. Does your surgery/rooms/unit currently collect any patient-reported outcome measures (PROMs) for bariatric patients as part of routine clinical practice?

Note: PROMs = questionnaires asking for the patients assessment of their outcomes of care. This may include measures of self-reported symptoms, functional status, health-related quality of life and psychosocial health.

|  | None If none, please go to question 7 | |
| --- | --- | --- |
|  | Yes, specifically for bariatric patients | If yes, please skip to question 8 |
|  | Yes, for all patients |  |

1. What is the main reason PROMs are not collected?

|  | Insufficient staff time or resources to follow-up on PROMs |
| --- | --- |
|  | Not aware of available PROMs |
|  | Unsure how to collect or use PROM data |
|  | PROMs not a local priority |
|  | PROMs not regarded as useful or impactful |
|  | Previously used PROMs have been discontinued |
|  | Planning to implement PROMs |
|  | Other *(please specify)* |
|  |  |

If you do not currently collect PROMs but do collect PREMs please skip to question 13

If you do not currently collect any PREMs or PROMs please skip to question 16

1. What are the main purposes for collecting PROMs?

(Please select all that apply)

|  | Mandated by health service |
| --- | --- |
|  | Monitoring and reviewing clinical practice |
|  | Screening surgical candidates |
|  | Directly informing patient care |
|  | Research purposes |
|  | Collected but not used |
|  | Other *(please specify)* |
|  |  |

1. Which PROM questionnaires does your surgery / rooms / unit currently use?

(Please select all that apply)

|  | BDI  (Beck Depression Inventory) |  | K10  (Kessler Psychological distress Scale) |  |
| --- | --- | --- | --- | --- |
|  | BODY-Q |  | PHQ-9  (Patient Health Questionnaire) |  |
|  | BQL  (Bariatric Quality of Life Index) |  | QOLOS  (Quality of Life for Obesity Surgery) |  |
|  | EQ-5D-5L |  | SF-36 |  |
|  | HADS  (Hospital Anxiety and Depression Scale) |  | TFEQ  (Three-Factor Eating Questionnaire) |  |
|  | IWQOL-Lite  (Impact of Weight on Quality of Life) |  | WHOQOL-BREF |  |
|  | Other  *(please specify)* |  | Surgery / Rooms / Unit-specific PROM  *(please outline what is assessed / measured)* | |
|  |  | | |  |

1. When do you collect PROMs?

|  | Pre-operative only | | |
| --- | --- | --- | --- |
|  | Post-operative only | | |
|  | Both pre- and post-operative |  |  |

1. How frequently do you collect PROMs **pre**-operatively?

|  | Once  *(Please specify time point)*…………………………………………………………………………………. | | |
| --- | --- | --- | --- |
|  | At regular intervals (e.g. 6 weeks, 6 months, annually)  *(Please specify)* …………………………………………………………………………………………………. | | |
|  | At each clinic appointment |  |  |
|  | Ad-hoc / as needed |  |  |
|  | Don’t collect PROMs pre-operatively | | |
|  | Other *(please specify)* |  |  |
|  |  | | |

1. How frequently do you collect PROMs **post**-operatively?

|  | Once  *(Please specify time point)*…………………………………………………………………………………. | | |
| --- | --- | --- | --- |
|  | At regular intervals (e.g. every 6 weeks, 6 months, annually)  *(Please specify)* …………………………………………………………………………………………………. | | |
|  | At each clinic appointment |  |  |
|  | Ad-hoc / as needed |  |  |
|  | Don’t collect PROMs post-operatively | | |
|  | Other *(please specify)* |  |  |
|  |  | | |

1. How are PRMs (PREMs and PROMs) administered?

(Please select all that apply)

|  | Pen & paper |  |  |
| --- | --- | --- | --- |
|  | Tablet or smart-phone | | |
|  | Web-based |  |  |
|  | Over the phone |  |  |
|  | Clinician / Nurse administered |  |  |

1. Is the collection of PRM data audited by your hospital or network?

|  | No |  |  |
| --- | --- | --- | --- |
|  | Yes – Process in place to review poor completion rates |  |  |
|  | Yes – No process in place to review poor completion rates |  |  |
|  | Unsure |  |  |

1. What action is taken based on PRM data?

|  | Reviewed by practice staff to inform clinical care |
| --- | --- |
|  | Aggregate data reviewed for benchmarking and/or quality assurance |
|  | None – collected but not used |
|  | Unsure |
|  | Other *(please specify)* |
|  |  |

1. Has your surgery/rooms/unit previously collected any PRMs that are now discontinued?

(Please select all that apply)

|  | None (please skip to question 18) |  | EQ-5D-5L |  |
| --- | --- | --- | --- | --- |
|  | AHPEQS |  | HADS |  |
|  | CAHPS Survey |  | IWQOL-Lite |  |
|  | PEECH |  | K10 |  |
|  | PEQ / PPE-15 |  | PHQ-9 |  |
|  | PSQ |  | QOLOS |  |
|  | BDI |  | SF-36 |  |
|  | BODY-Q |  | TFEQ |  |
|  | BQL |  | WHOQOL-BREF |  |
|  | Other *(please specify)* |  | Surgery/Room/Unit-specific PRM  *(please outline what was assessed/measured)* | |
|  | | | |  |

1. What are the main reasons for discontinuation?

(Please select all that apply)

|  | Measures were too long / had too many questions |  |  |
| --- | --- | --- | --- |
|  | Patients complained about completing PRMs |  |  |
|  | Collected data was incomplete |  |  |
|  | Data was not useful for clinical practice |  |  |
|  | Associated cost / licence fee of PRM |  |  |
|  | Insufficient resources to collate / analyse information |  |  |
|  | Updated to a different PRM or PRMs |  |  |
|  | Other *(please specify)* |  |  |
|  |  | | |

**SECTION TWO**

1. The Bariatric Surgery Registry is currently developing PRMs for long-term patient monitoring, including quality of life and psychosocial health data collection from patients within the registry.

Do you think this data will be useful?

|  | Yes | |  |  |
| --- | --- | --- | --- | --- |
|  | No | If no or unsure, please provide any reasoning as to  why you think this data may not be useful | | |
|  | Unsure |  |  |  |
|  | | | | |

1. Below are several applications of registry-based PRM data. Please rank these applications from 1 – 6 based on how useful you believe each application would be to routine clinical practice (with 1 being most useful);

| A screening tool  *(e.g. screening for depression or anxiety)* |  | |
| --- | --- | --- |
| A monitoring tool  *(i.e. monitoring patient progress following surgery)* |  |  |
| Promoting shared decision making  *Alerting the clinician to the patient’s health concerns and priorities* |  |  |
| A decision aid  *(i.e. using aggregated PRM data to help patients and clinicians make treatment decisions based on outcomes of similar patients)* |  |  |
| Facilitating communication amongst multi-disciplinary teams  *Assisting clinicians to work towards achieving common treatment goals and involve patients in setting these goals* |  |  |
| Evaluating effectiveness and quality of care  *Using aggregated PRM data to evaluate or compare surgical procedures and service providers* |  |  |

1. Below are some potential outcomes of registry-based PRM collection. Please rank these outcomes from 1 – 5 based on how important you believe each outcome would be for routine clinical practice (with 1 being most important);

| Reduced strain on staff time / resources |  |
| --- | --- |
| Enhanced patient – physician communication  *(e.g. facilitating discussion about quality of life and mental health)* |  |
| Increased reporting and recognition of symptoms  *(e.g. symptom monitoring and alerts)* |  |
| Increased understanding of patient health needs and QoL |  |
| More actions taken based on PRM data  *(e.g. increased number of referrals to mental health professionals)* |  |

1. How often do you think PRM data should be collected by the BSR?

|  | Once  *(Please specify time point)*…………………………………………………………………………………. | | |
| --- | --- | --- | --- |
|  | At regular intervals (e.g. every 6 weeks, 6 months, annually)  *(Please specify)* …………………………………………………………………………………………………. | | |
|  | At set time points (e.g. at 6 weeks, 6 months, and annually thereafter)  *(Please specify)* | | |
|  |  | | |
|  | Other *(please specify)* |  |  |
|  |  | | |

1. Do you have any other comments to add about collecting and using PRMs either in general or through the Bariatric Surgery Registry?

|  |  |
| --- | --- |

1. Would you be interested in collaborating with the Bariatric Surgery Registry on their developing research into the development and implementation of PRMs?

|  | Yes |  |  |
| --- | --- | --- | --- |
|  | No |  |  |
|  | Unsure |  |  |

Thank you for your contribution to this survey
